# The role of gel-phase domains in electroporation of vesicles

**DOI:** 10.1038/s41598-018-23097-9

**Published:** 2018-03-19

**Authors:** Dayinta L. Perrier, Lea Rems, Michiel T. Kreutzer, Pouyan E. Boukany

**Affiliations:** Department of Chemical Engineering, Delft University of Technology, 2629 HZ Delft, The Netherlands

## Abstract

Transient permeabilisation of the cell membrane is a critical step to introduce drugs or DNA into living cells, yet challenging for both biological research and therapeutic applications. To achieve this, electroporation (or electropermeabilisation) has become a widely used method due to its simplicity to deliver almost any biomolecule to any cell type. Although this method demonstrates promise in the field of drug/gene delivery, the underlying physical mechanisms of the response of the heterogeneous cell membrane to strong electric pulses is still unknown. In this study, we have investigated the role of gel-phase lipids in the electroporation of binary giant unilamellar vesicles (GUVs), composed from DPPC (gel-phase) and DPhPC (fluid-phase) lipids (molar ratio 8:2 and 2:8). We have observed that the exposure to electric pulses leads to expel of fluid-phase lipids and concomitant decrease in GUV size, whereas the gel-phase domains become buckled. Based on experiments on pure fluid-phase and gel-phase GUVs, we have found that fluid-phase lipids can be expelled by electrical forces and the highly viscous gel-phase lipids cannot. Moreover, our analyses suggest that pore formation occurs primarily in fluid-phase domains and that the pore size is similar in all GUVs containing fluid-phase lipids, irrespective of the gel-phase percentage.

## Introduction

Every living cell is enclosed by the cell membrane, which provides a protective barrier and governs the cell permeability to the extracellular environment. The cell membrane consists mainly of lipids, that self-assemble based on hydrophobic and hydrophilic interactions. In order to permeabilise the lipid bilayer for drug delivery, these interactions must be overcome. By applying a direct current (DC) pulse the membrane can be permeabilised transiently, due to the transmembrane voltage that builds up on the membrane during the pulse^1^. Transient pores are formed when the transmembrane voltage exceeds a critical value, typically between 0.2 and 1 V^2,3^. This approach, referred to as electroporation or electropermeabilisation, is used for both living cells and lipid vesicles to enhance the transmembrane transport of drugs, genetic material, and other (bio)molecules in the field of medicine, food processing, and environmental applications^4–7^. Despite the wide use of this technique, the cascade of mechanisms that are operative during the electric pulse is still not fully understood.

To reveal the electroporation mechanisms, electroporation is studied using simplified model systems such as giant unilamellar vesicles (GUVs)^8–10^, planar lipid bilayers^11^ and different theoretical methods including molecular dynamics (MD) simulations^12–14^. GUVs provide the benefit of isolating the function of the membrane from the complex intracellular components involved in a cell, while resembling the size of the cell and curvature of the cell membrane. Additionally, the composition of the membrane is controllable, for example the GUVs can be prepared from lipids with different phase-states. Depending on their molecular structure, the lipids can be either in the fluid-phase state, containing high mobility and low order, or in the gel-phase state, which contain a low mobility and are tightly packed^15^. Experiments on GUVs have revealed that electroporation depends strongly on the phase-state of the lipids. It has been shown that electroporation of fluid-phase GUVs is associated with formation of micrometre-sized pores (macropores) and lipid loss^16–18^. Compared to fluid-phase GUVs, Knorr *et al*.^19^ have shown that gel-phase GUVs electroporate at considerably higher critical transmembrane voltage of about 10 V, which they have attributed to a higher bending rigidity and thickness of the gel-phase membrane. Furthermore, they have observed irreversible cracks in electroporated gel-phase GUVs, in contrast to the reversible macropores in fluid-phase GUVs. Mauroy *et al*.^20^ have demonstrated that the phase state plays the decisive role in the increased critical transmembrane voltage of gel-phase GUVs with respect to fluid-phase GUVs, and not the carbon chain length, due to the large cohesion of the gel-phase lipids. This increased critical transmembrane voltage for electroporation of gel-phase lipids is supported by MD simulations^12^.

Most of the studies discussed above have been performed on membranes consisting of single-phase lipids. However, the cell membrane is composed of various types of lipids which can coexist in different phases, organised in domains^21,22^. Studies investigating electroporation of multiphase membranes are scarce. Liu *et al*.^23^ have performed experiments on large unilamellar vesicles (diameter ~1 *μ*m) with fluid-phase and gel-phase lipids and have demonstrated that the critical transmembrane voltage increases by increasing the amount of gel-phase lipids in the vesicles. Reigada^13^ and Van Uitert^11^ have studied heterogeneous lipid membranes consisting of fluid-ordered and fluid-disordered phases by MD simulations and resistance measurements on lipid bilayers, respectively. By mixing cholesterol into a binary lipid bilayer, the two lipids can organize into fluid-ordered (containing the majority of the cholesterol) and fluid-disordered domains^24^. Both studies^11,13^ have shown that the fluid-ordered domains increase the critical transmembrane voltage. In addition, the MD simulations have given the molecular insight that the pores are preferentially formed in the fluid-disordered domains^13^. These results are supported by Sengel and Wallace^25^ with experiments on droplet-interface bilayers.

In this study we focus on the role of gel-phase domains in electroporation, by mixing gel-phase and fluid-phase lipids in GUVs with a radius between 10 and 30*μ*m. DPhPC (1,2-diphytanoyl-sn-glycero-3-phosphocholine) and DPPC (1,2-dipalmitoyl-sn-glycero-3-phosphocholine) are used as the fluid-phase and the gel-phase lipids at room temperature, respectively. These two lipids contain the same head group and differ in their carbon chain: DPhPC has methyl groups (fluid-phase) and DPPC has a linear chain (gel-phase). GUVs with four different lipid compositions have been prepared, going from pure fluid to pure gel-phase GUVs with two intermediate binary phase mixtures. All GUVs have been exposed to multiple electric pulses of increasing electric field strength, and their responses have been imaged using bright-field microscopy.

## Results

**Figure 1:**
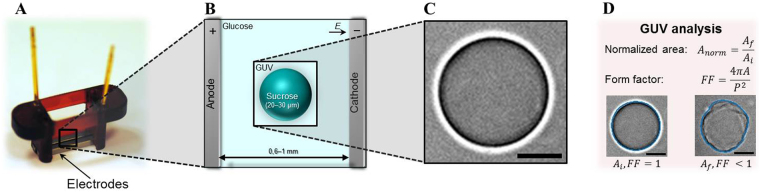
The experimental setup and analysis for the electroporation of the GUVs. (**A**) A picture of the electroporation setup. (**B**) A schematic of the sucrose-filled GUV in the glucose environment, in between the electrodes with a distance ranging between 0.6 and 1 mm. The drawing is not to scale. (**C**) Bright field image of a sucrose-filled GUV in a glucose environment. (**D**) The analysis of the GUVs: the contour is tracked, after which the area, *A*, and the perimeter, *P*, are determined. From these, the normalised area and the form factor are calculated. The form factor is 1 for a circular GUV, and less than one for a non-circular GUV. The scale bar is 10 *μ*m.

The responses of the GUVs have been studied before, during and after the electric field application. The interior of the GUVs consists of 200 mM sucrose, whereas the exterior consists of 200 mM glucose to create a difference in both the density and the refractive index. Consequently, the density difference has enabled the GUVs to sediment to the bottom of the glass slide, and the mismatch in refractive index has enabled the imaging of the GUVs. The setup is shown in Fig. 1A–C. Unless noted otherwise, each GUV has been exposed to multiple 500-*μ*s-long rectangular electric pulses with increasing amplitude, so each subsequent pulse results in a higher electric field strength in between the electrodes. The time interval between the pulses is approximately 5 minutes, to minimize the effect of the former pulse. Therefore, these pulses are referred to as individual 500 *μ*s pulses. Nevertheless, it should be noted that the effects of consecutive pulses could be accumulating during the experiment. The response of the GUV is captured by imaging at approximately 10 to 25 frames per second. The contour of the GUV before, during and after the electric pulse application has been tracked, extracting the area and the perimeter of the GUV with a pixel count, as depicted in Fig. 1D. These properties have been used to determine the shrinkage of the GUV induced by each applied pulse, by calculating the normalised area (*A*_*norm*_ = *A*_*f*_/*A*_*i*_) before (*A*_*i*_) and after (*A*_*f*_) the application of the pulse. In addition, the shape of the GUV has been determined based on the form factor (*FF*):

1 $$FF=\frac{4\pi A}{{P}^{2}}$$

where *A* represents the area of the GUV, and *P* the perimeter. The form factor is 1 for a circular shape, and less than 1 when the shape is non-circular. Apart from the area loss and form factor, loss of contrast after the electric pulses has been used to indicate electroporation of the membrane.

### Fluid-phase and gel-phase GUVs

**Figure 2:**
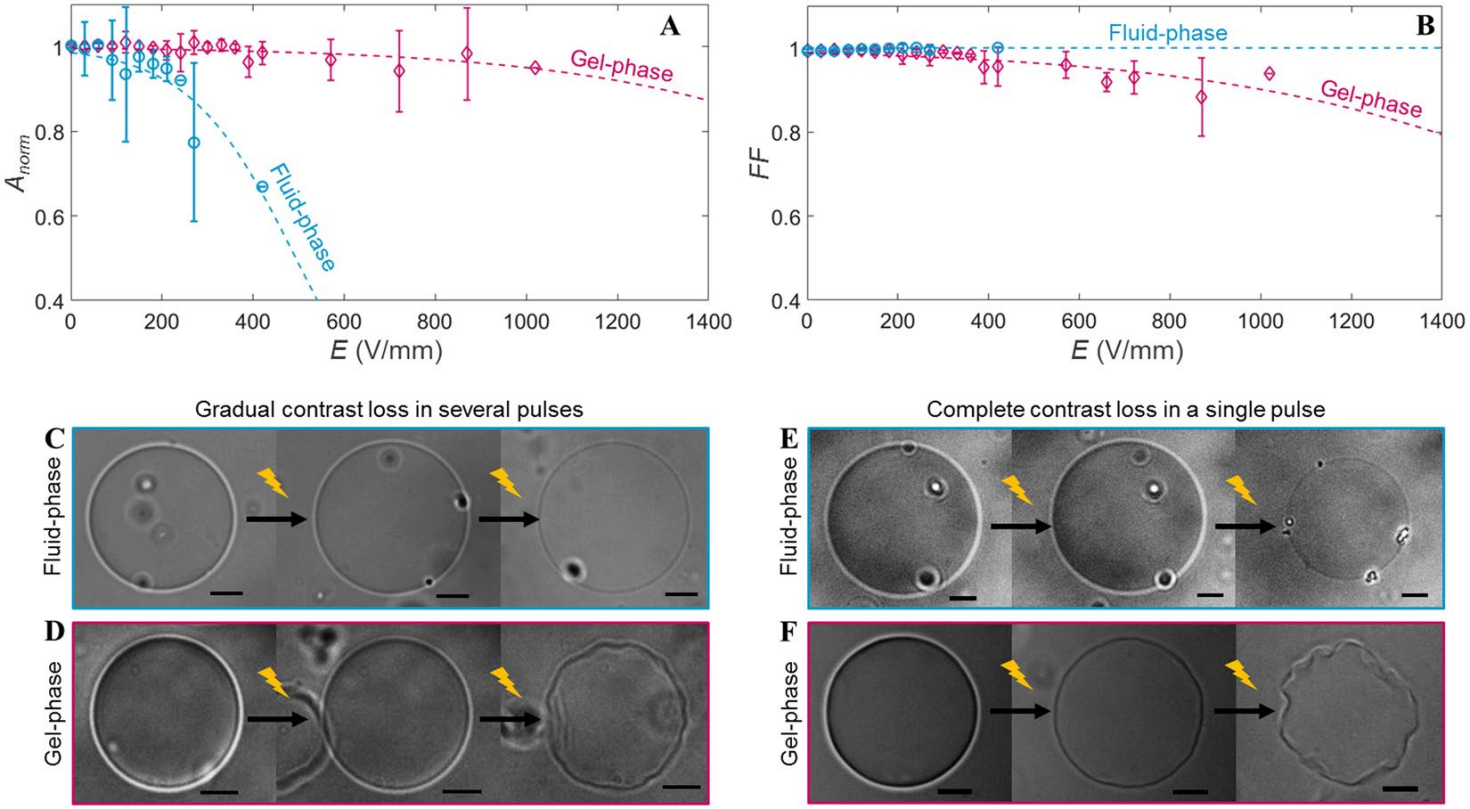
The response of the fluid-phase (in blue) and gel-phase (in pink) GUVs to the electric field. (**A**) and (**B**) show, respectively, the averaged normalised area and form factor plotted versus the electric field of the pulse. The fluid-phase data is averaged over 11 different GUVs, with radii between 11 and 28 *μ*m. The gel-phase data is averaged over 12 different GUVs, with radii between 10 and 22 *μ*m. The error bars in the plots represent the standard deviation. The dotted lines represent the least-squares fit with a sigmoid curve. (**C**) and (**D**) are snapshots of the fluid-phase and gel-phase GUVs showing the gradual contrast loss. (**C**) From left to right: before pulse application, more than 100 seconds after the tenth pulse at 52 V/mm and after the twentieth pulse at 100 V/mm. (**D**) From left to right: before pulse application, more than 100 seconds after the fourth pulse at 74 V/mm and after the twenty-fourth pulse at 372 V/mm. (**E**) and (**F**) are snapshots of the fluid-phase and gel-phase GUVs showing the complete contrast after one of the pulses. (**E**) From left to right: before pulse application, more than 100 seconds after the second pulse at 89 V/mm and after the third pulse at 97 V/mm. (**F**) From left to right: before pulse application, more than 100 seconds after the third pulse at 595 V/mm and after the fourth pulse at 744 V/mm. The scale bar in all images is 10 *μ*m.

**Figure 3:**
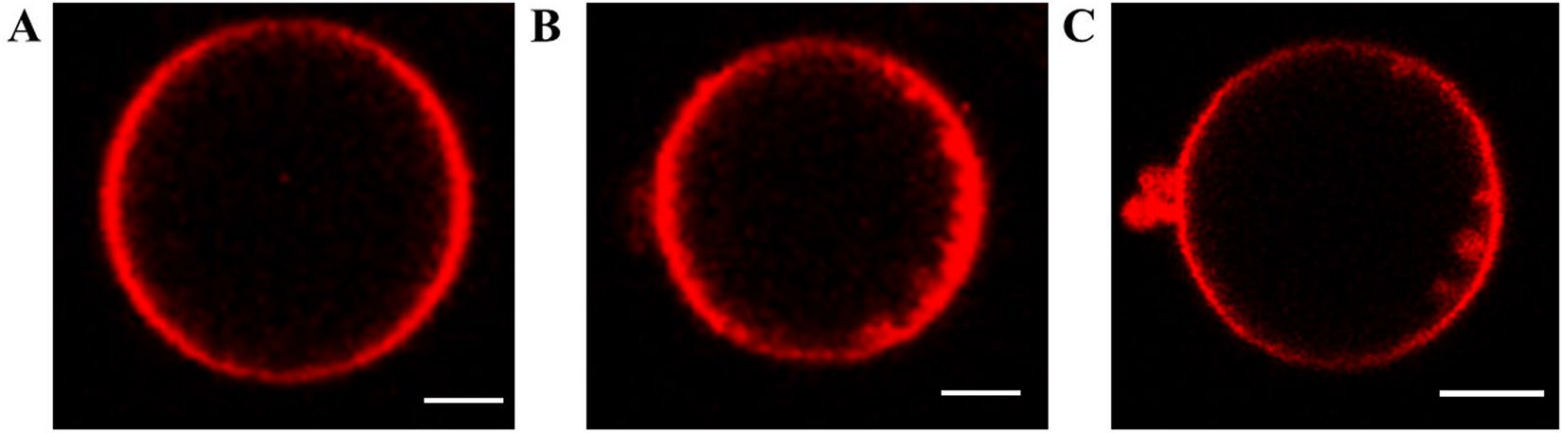
A DPhPC GUV (**A**) before pulse application and two representative examples of the shrinkage mechanism of the DPhPC GUVs (**B**) showing tubule formation (**C**) and vesicle formation. In these examples, the GUVs have been exposed to 2–8 pulses, duration 5 ms, amplitude 90 V/mm. The majority of the GUVs have exhibited vesicle formation. The scale bar in all images is 5 *μ*m, the cathode is on the left side and the anode on the right side of the GUVs.

In order to understand the role of lipid phase-state on the response of GUVs to electric pulses, we have first studied the response of pure fluid-phase and pure gel-phase GUVs. In agreement with previous observations on fluid-phase egg phosphatidylcholine and DOPC (1,2-dioleoyl-sn-glycero-3-phosphocholine) GUVs^16,26^, the fluid-phase DPhPC GUVs have exhibited shrinkage as a consequence of the electric pulses (Fig. 2A). The GUVs have decreased in size up to ~30% when reaching the highest electric field tested (~400 V/mm). The gel-phase DPPC GUVs also exhibited a slight shrinkage at higher electric field (≥600 V/mm), confirming that the gel-phase GUVs are more stable than fluid-phase GUVs, as observed previously^19,23^. However, a difference in the shrinkage mechanism for the fluid-phase and the gel-phase GUVs can be seen from the change in the form factor. The form factor of the fluid-phase GUVs remains around one, indicating that the GUVs remain spherical (Fig. 2B). This is due to the simultaneous loss of the surface area and the interior volume. To visualise the shrinkage mechanism of the fluid-phase GUVs, confocal measurements have been conducted. In addition to exposing the GUVs to multiple 500 *μ*s pulses, the GUVs were also exposed to multiple 5 ms pulses of 0.33 Hz to compare the shrinkage mechanism to the former study of Portet *et al*.^16^. It is found that the DPhPC fluid-phase GUVs exhibit shrinkage due to either small vesicle formation and/or tubulation (Fig. 3), as observed previously^16^ although less pronounced. The vesicle formation is the dominant phenomenon. After expulsion, both the small vesicles and tubules remain attached to the GUV. No reabsorption of neither the vesicles nor the tubules is observed on the time scale of the experiments (2 min). This shrinkage mechanism is in this work referred to as lipid loss or lipid expel, despite the fact that the formed daughter vesicles and tubules remain attached to the mother GUV. On the contrary to the fluid-phase GUVs, gel-phase GUVs have shown no apparent loss of lipids (Figure S1). Moreover, the form factor of the gel-phase GUVs has dropped below one, showing that these GUVs have lost their spherical shape. On the application of pulses with an electric field strength ≥400 V/mm, the gel-phase GUVs have deformed permanently into asymmetrical shapes, here referred to as buckling. This buckling behaviour of the gel-phase GUVs demonstrates that these GUVs have lost part of their interior volume while conserving their lipids.

To assess the permeability of the GUVs after pulse application, the contrast loss of the GUVs is studied. Two different contrast loss regimes can be observed. About 50% of the tested fluid-phase GUVs have expressed a gradual contrast loss throughout the whole experiment, where consecutive pulses of increasing amplitude have been applied (Fig. 2C). At higher fields (≥100 V/mm), the cumulative contrast loss is accompanied by shrinkage of the GUVs. These GUVs have shrank rapidly (within ~100 ms during/after the pulse) by up to few percent. These results are similar as reported before on other fluid-phase GUVs like DOPC^26^. Similarly, gradual contrast loss is observed of about 50% of the gel-phase GUVs at lower electric fields, followed by gradual buckling at higher fields (Fig. 2D). However, in about 50% of the tested fluid-phase GUVs, we have also observed a complete drop in contrast after one individual pulse with a sufficient amplitude. The complete contrast loss after this pulse takes place for tens of seconds, and is in half of these cases accompanied by more profound (up to ~30%) decrease in the GUV size (Fig. 2E and Movie S1). Therefore, it appears that an electric pulse can lead to both a short term and a long term permeability of the fluid-phase GUVs. The gel-phase GUVs also display long term permeability of the membrane after an individual pulse (~50% of the gel-phase GUVs), similarly to the fluid-phase GUVs. In gel-phase GUVs, the contrast loss is accompanied by buckling of the GUV instead of lipid loss (Fig. 2F and Movie S2).

In addition to the duration of the membrane permeability after the pulse application, the movies also provide the dynamics of the GUVs immediately after the pulse and during the relaxation. Similarly to earlier studies, elliptical deformations and macropores have been observed in fluid-phase GUVs. For the gel-phase GUVs first reversible electroporation is observed and at higher electric fields folds and cracks have been seen immediately after the pulse application, similar as reported by Knorr *et al*.^19^. However, our gel-phase GUVs slowly relax back into a buckled shape after approximately 170 seconds (Movie S3). These slow responses have not been reported in former studies, possibly because of the duration of their experiments.

### Binary GUVs containing fluid-phase and gel-phase lipids

**Figure 4:**
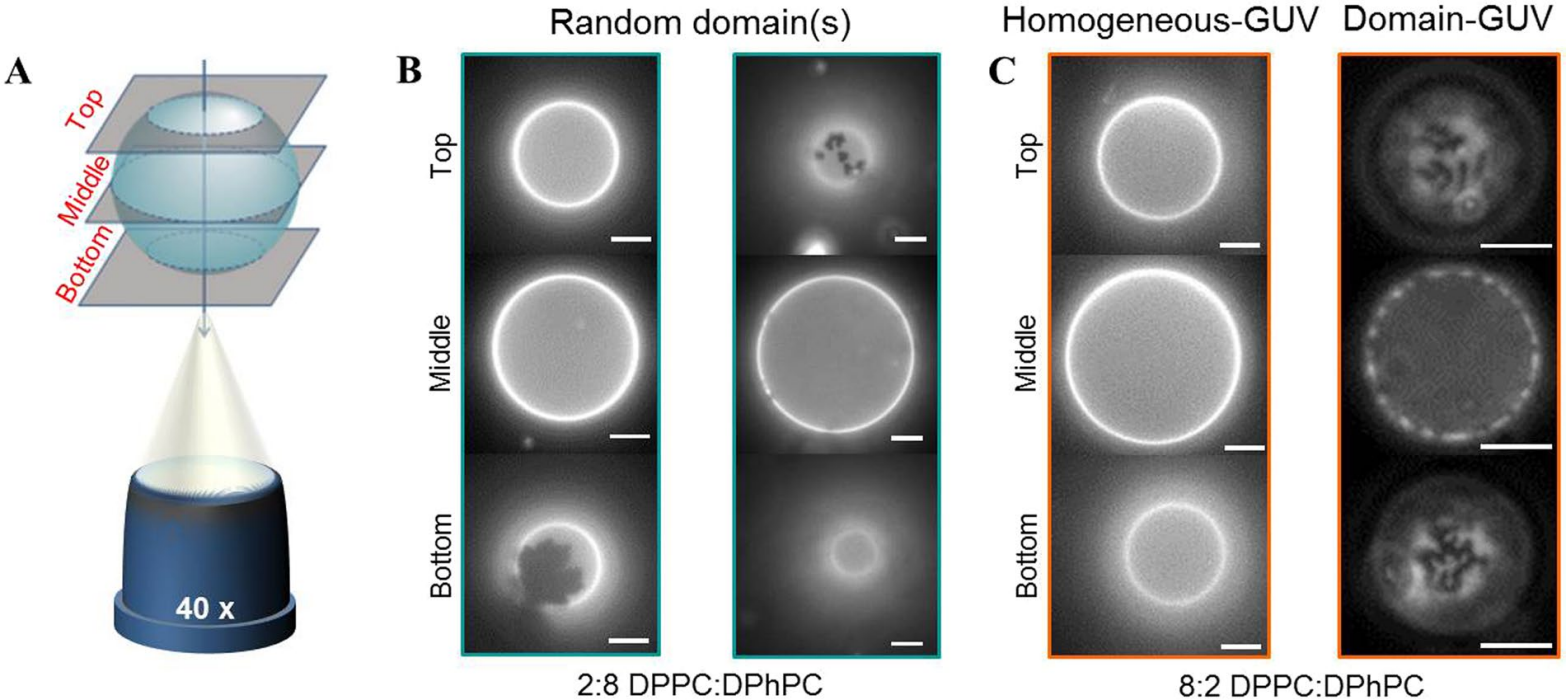
The fluorescence images of the binary GUVs before electroporation. (**A**) A schematic drawing of the three different focal planes (bottom, middle and top planes) of the GUVs, not drawn to scale. (**B**) Two examples of the 2:8 DPPC:DPhPC GUVs, showing different gel-phase domains. The GUVs are visualised by use of fluorescence microscopy, where the dark parts represent the gel-phase domains, and the bright parts the fluid-phase or mixed domains. (**C**) The two different categories of the 8:2 DPPC:DPhPC GUVs. Left: a homogeneous-GUV, showing no visible domains. Right: a domain-GUV, showing a uniform distribution of dark gel-phase domains on the surface of the GUV. The scale bar is 10 *μ*m.

To assess the influence of gel-phase domains in a fluid-phase membrane on the GUV response we have prepared binary GUVs with two different molar ratios of DPPC:DPhPC, (i) 2:8 and (ii) 8:2. The mixing behaviour of the lipids in the GUV membrane has been determined by imaging GUVs in three different focal planes using fluorescence microscopy, as schematically depicted in Fig. 4A. The GUVs are fluorescently labelled with 1,2-dioleoyl-sn-glycero-3-phosphoethanolamine-N-(lissamine rhodamine B sulfonyl) (ammonium salt) (DOPE-RhoB) lipids, which preferentially organise in the fluid-phase domains; therefore the fluid-phase and gel-phase domains can be visualised as bright and dark patches, respectively, in the fluorescence images. The phase-separation of the two phases is governed by the cooling rate of the GUVs from a temperature above the transition temperature of the gel-phase lipids, where the GUVs are in a one-phase state, to a temperature below the transition temperature, where two coexisting phases can be formed^27,28^. During the cooling of the GUVs the gel-phase domains nucleate and grow as a function of cooling rate, resulting in phase-separation^29^. The size, shape and number of domains are dependent on the ratio of the two lipids^27^, the cooling down rate^29^ and the membrane tension^30^. Based on the mixing behaviour, these binary GUVs have been divided into three different categories: (1) the 2:8 DPPC:DPhPC GUVs, which exhibit few gel-phase domains distributed stochastically in the size and number (Fig. 4B), (2) the 8:2 DPPC:DPhPC GUVs where no gel-phase domains were visible, which suggests that the lipids are homogeneously mixed, referred to as homogeneous-GUVs (Fig. 4C), and (3) the 8:2 DPPC:DPhPC GUVs showing phase separation by displaying gel-phase domains, called domain-GUVs (Fig. 4C). These three categories provide the opportunity to study the influence of the distribution of the gel-phase domains on the GUV response to the electric field. It must be noted that the domains are stable throughout the whole experiment, also after pulse application.

**Figure 5:**
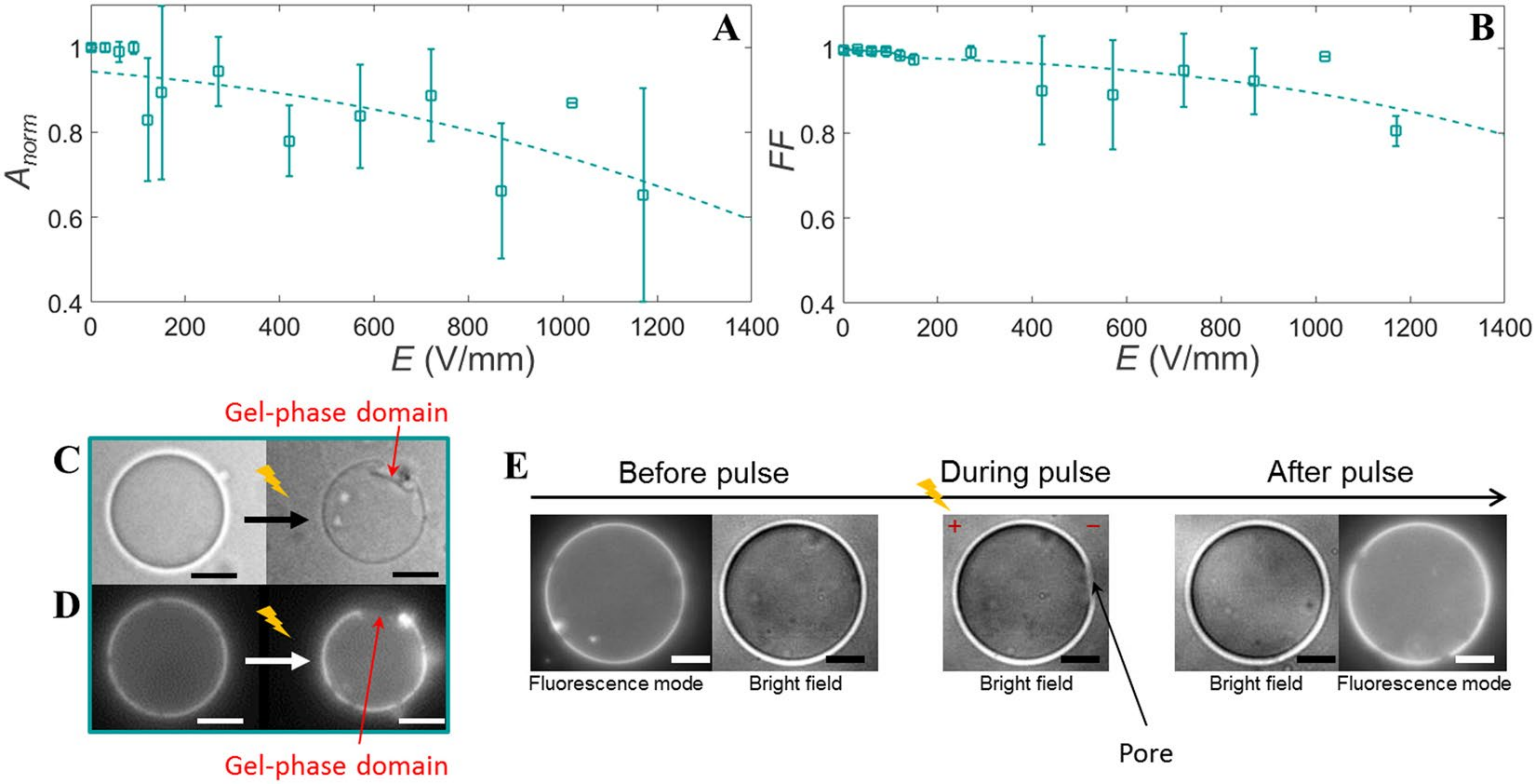
The normalised area and the form factor of the 2:8 DPPC:DPhPC GUVs. (**A**) The averaged normalised area and (**B**) the form factor of the 2:8 DPPC:DPhPC GUVs. The data is averaged over 13 different GUVs, with radii between 11 and 26 *μ*m. The dotted lines represent the least-squares fit with a sigmoid curve. The error bars in the plots represent the standard deviation. (**C**) Images of a GUV before and more than 100 seconds after the second pulse at 445 V/mm pulse. (**D**) Fluorescence images of the same GUV. The fluorescence image after the pulse shows that the buckled patch is located at the gel-phase domain (the dark patch). (**E**) Bright field and fluorescence images of a GUV before the pulse, during the pulse when a macropore is observed, and after the third pulse at 29 V/mm. The fluorescence images are only captured before and after the pulse. The scale bar is 10 *μ*m.

The 2:8 DPPC:DPhPC GUVs enable to capture the role of small, stochastically distributed gel-phase domains on the response of the GUVs to an electric pulse. Similarly to the pure fluid-phase GUVs, these 2:8 DPPC:DPhPC GUVs show a shrinkage in size at an electric field above ~150 V/mm (Fig. 5A). At higher fields (~400 V/mm) the form factor drops, indicating the 20% of DPPC lipids cause a small buckling effect (Fig. 5B). The fluorescence images after pulse application reveal that the buckled patches are located at the gel-phase domains (Fig. 5C and D). Nevertheless, no correlation is found between the gel-phase domains distribution and either the area loss or the form factor, due to the stochastic distribution of the gel-phase domains. The captured movies also reveal some of the dynamics of the 2:8 DPPC:DPhPC GUVs. Pores have been observed for seven GUVs out of the thirteen captured GUVs, as shown in the snapshots in Fig. 5E and in Movie S4. These pores are found both on the cathode and the anode side. Due to the limited temporal resolution, the pores are only visible for approximately one single frame. Therefore, only the presence of pores can be confirmed and the maximum pore size cannot be determined. These pores are similar to earlier reported pores in fluid-phase GUVs^31,32^ and are referred to as macropores. In addition, all captured GUVs have exhibited complete contrast loss after one of the individual pulses. This indicates that the electric field causes long term defects in the binary membrane.

**Figure 6:**
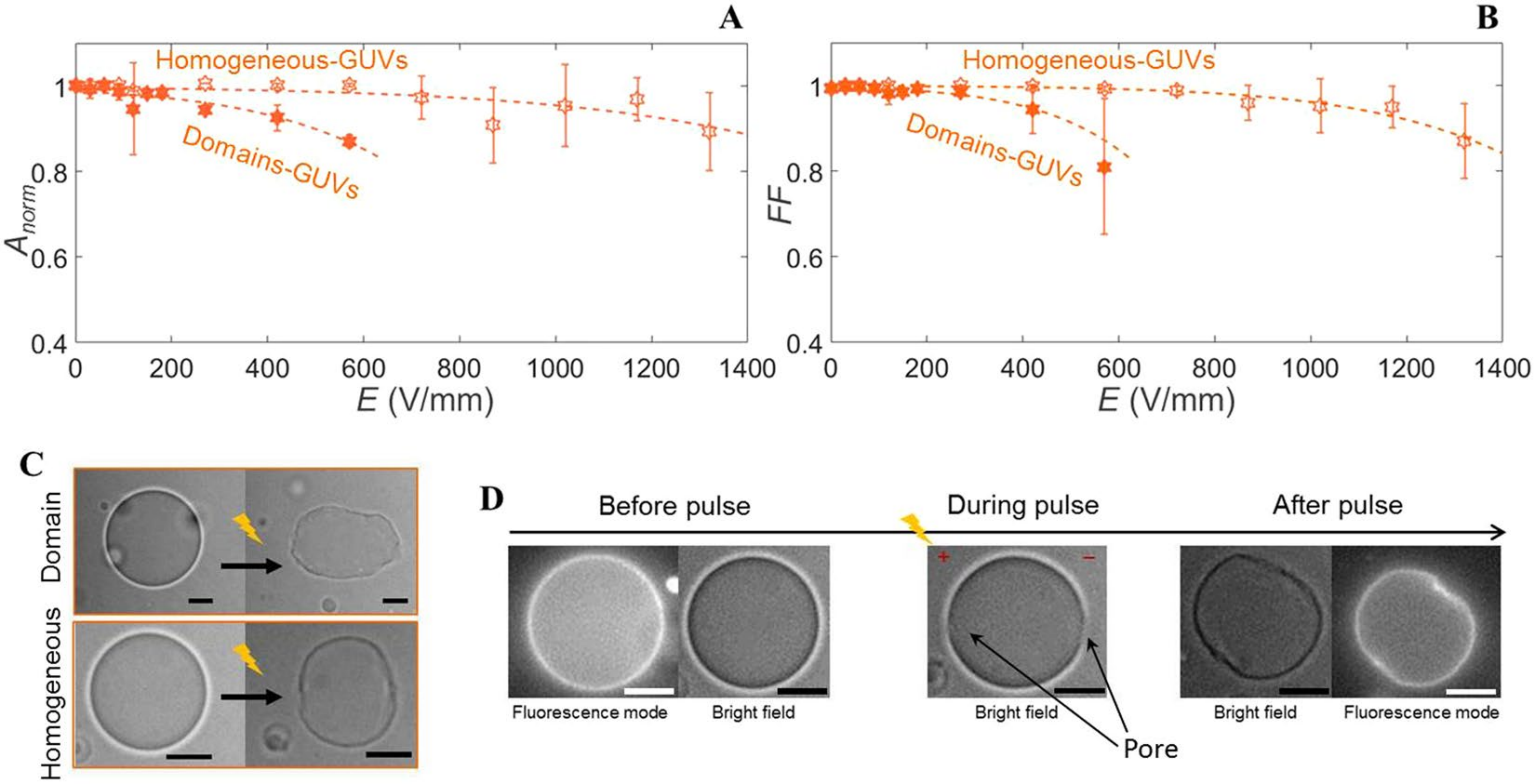
The response of the 8:2 DPPC:DPhPC GUVs to increasing electric pulses. (**A**) The averaged normalised area and (**B**) the form factor of the homogeneous- and domain-GUVs. The data is averaged over 13 different GUVs, with radii between 11 and 28 *μ*m, where 7 GUVs contain domains (radius between 11 and 28 *μ*m) and 6 GUVs have fully mixed phases (radius between 14 and 22 *μ*m). The dotted lines represent the least-squares fit with a sigmoid curve. (**C**) The images of the domain- and the homogeneous-GUVs before and more than 100 seconds after the first pulse at 445 V/mm (domain-GUV) or the third pulse at 890 V/mm (homogeneous-GUV), respectively. (**D**) Bright field and fluorescence images of the homogeneous-GUV shown in (**C**) before the pulse, during the pulse when a macropore is observed, and after the pulse. The fluorescence images are only captured before and after the pulse. The scale bar is 10 *μ*m.

The 8:2 DPPC:DPhPC GUVs have been divided into two different categories, the homogeneous-GUVs and the domain-GUVs, to reveal the influence of the mixing behaviour of the two lipids on the GUV response. The homogeneous-GUVs have shown a similar size decrease as the pure gel-phase GUVs (Fig. 6A), whereas the domain-GUVs have displayed size decrease at a lower electric field. The same holds for the drop in the form factor of the homogeneous- and the domain-GUVs (Fig. 6B). This indicates that the lipids in the homogeneous-GUVs are predominantly in the gel-phase. Despite the different electric field strengths required, the buckling behaviour of both types of GUVs appears similar (Fig. 6C). The dynamics of the domain-GUVs and the homogeneous-GUVs show differences in their response to the electric pulse. As the homogeneous-GUVs relax back into a quasi-spherical, buckled shape, the domain-GUVs can also relax back into a quasi-elliptical, buckled shape (Movie S5 and S6). For two of the six homogeneous-GUVs macropores have been observed both on the anode and the cathode side (Fig. 6D and Movie S5). The lack of observation of macropores for the remaining GUVs may be caused by the limited temporal resolution of the measurements. Finally, about ~80% of the homogeneous-GUVs and ~70% of the domain-GUVs exhibit complete contrast loss after one of the individual pulses.

## Discussion

The binary GUVs exhibit a complex behaviour with characteristics of both fluid-phase and gel-phase lipids. The shrinkage of these GUVs is associated with tubulation and/or vesicle formation observed for the fluid-phase GUVs and the buckling at higher electric fields is related to the gel-phase lipids. In order to explain this mixed behaviour of the binary GUVs, we will first shed light on why fluid-phase GUVs expel lipids and gel-phase GUVs do not. Consequently, this understanding of the pure-phases will enable us to provide a mechanistic insight into the response of the binary GUVs to the electric pulses.

The decrease in size of the fluid-phase GUVs is due to loss of membrane lipids via formation of small membrane vesicles and/or tubules. Tubulation has been observed when exposing GUVs to both DC and AC electric field^16,33^. The mechanisms of this lipid loss are not well understood yet. During the pulse, the electric stress builds up due to the ions accumulated on both sides of the membrane. Small curvatures in the bilayer lead to a locally unbalanced electric stress and membrane shape undulations because of the different surface charge densities on each side of a curved membrane^34^. This unbalanced stress is directly proportional to the membrane surface tension induced by the electric field, which is given by^33–36^

2 $${\sigma }_{el}\,\approx \,0.5{C}_{m}{{\rm{\Psi }}}_{m}^{2}$$

where *C*_*m*_ is the membrane capacitance and Ψ_*m*_ is the transmembrane voltage. For a spherical GUV, the maximum Ψ_*m*_ can be calculated as^37^

3 $${{\rm{\Psi }}}_{m}=1.5RE\mathrm{(1}-\exp (-t/\tau ))$$

where *R* is the radius of the GUV, *E* is the applied electric field strength, *t* is the duration of the electric field, and *τ* is the characteristic membrane charging time given by

4 $$\tau \approx R{C}_{m}(\frac{1}{{\lambda }_{e}}+\frac{1}{{\lambda }_{i}})$$

where *λ*_*e*_ and *λ*_*i*_ are the external and the internal conductivities of the liquids, respectively. Antonova *et al*.^33^ recently have proposed a model, which considers that the electrical tension *σ*_*el*_ exerts a force tending to form a small membrane bud and elongate it into a membrane tubule, analogous to the force required to mechanically pull a membrane tubule (tether) from a GUV. This is based on their experimental findings of tubule growth in phosphatidylcholine GUVs subjected to non-electroporative AC fields (1–2 kHz) with a strength beyond ~15 V/mm. They have estimated the force which triggers the tubule formation as^33^:

5 $${F}_{el}=2\pi \sqrt{2{k}_{c}{\sigma }_{el}}$$

where *k*_*c*_ is the bending elasticity modulus. In order for a tubule to grow, this *F*_*el*_ needs to be compared to the opposing force due to the viscous dissipation accompanying the tubule formation. This dissipation is produced by the slipping motion between the two leaflets of the lipid bilayer and by the surface stress due to the extensional flow of the membrane from the GUV body to the tubule^38,39^:

6 $${F}_{v}=2\pi {\eta }_{eff}{V}_{t}$$

where *η*_*eff*_ represents the effective viscosity, which includes the membrane surface viscosity and the intermonolayer slip coefficient, and *V*_*t*_ represents the velocity of tubule elongation^39^. The estimates of the electrical (*F*_*el*_) and viscous (*F*_*v*_) forces can shed light on why lipid loss cannot be observed in gel-phase GUVs. As the surface viscosity of gel-phase lipids is about six orders of magnitude higher than the viscosity of fluid-phase lipids^40^, we expect that these lipids cannot be removed by the electric field due to the high viscous forces of the gel-phase lipids, whereas the fluid-phase lipids can be removed in the form of tubulation and vesicle formation.

The electrical and viscous forces from above indicate that the binary GUVs mainly expel their fluid-phase lipids and keep their gel-phase lipids. This is supported by the observations that buckling takes place at higher electric fields for the 2:8 DPPC:DPhPC GUVs, after which many of the fluid-phase lipids are already removed by the electric field. Moreover, the expel of fluid-phase lipids is supported by the dynamics of the domain-GUVs. Such GUV can be elongated during the pulse. Yet, during the relaxation, after the fluid-phase lipids have been removed, the GUV appears more rigid and thus remains permanently elliptically shaped (Movie S5 and S6). This phenomenon is observed for domain-GUVs, though such behaviour is not observed for the homogeneous-GUVs.

Both the 2:8 and the 8:2 DPPC:DPhPC GUVs have revealed macropores, even for the homogeneous-GUVs. As the pores are similar to the ones observed in the pure fluid-phase GUVs, these pores indicate that the pore formation takes place in the fluid-phase domains. This is corroborated by MD simulations^13^ and experiments on droplet-interface bilayers^25^, that have shown a preferential pore formation in the fluid-phase domains for heterogeneous systems. Additionally, the macropores in the 8:2 DPPC:DPhPC GUVs show that large (observable) pores can be formed, despite the high concentration of DPPC lipids. During the exposure of a GUV to an electric pulse the pressure inside the GUV increases due to an increase in the surface tension of the membrane. As soon as the pores are formed, the built-up surface tension can be released both by the expansion of pores in the membrane and the efflux of the internal liquid through the expanding pores^31,41^. Hence, by determining the efflux, we can gain insight on the sizes of the pores. For simplicity, we assume that the majority of the efflux is transported through a single pore. This is supported by former research, where a single macro-pore has been observed^16–18^ in electroporated GUVs. Under this assumption the efflux *Q* can be directly related to the pore radius *r*^41,42^:

7 $$Q=\frac{2\sigma }{3{\eta }_{0}R}{r}^{3}$$

where ${\eta }_{0}$ is the liquid viscosity, *σ* is the membrane surface tension. From our experiments we can extract the efflux from the observed area loss *A* = *πR*^2^ of the GUV as follows:

8 $$Q\,=\,-\frac{dV}{dt}\,=\,-4\pi {R}^{2}\frac{dR}{dt}\,=\,-4A\frac{d}{dt}(\sqrt{\frac{A}{\pi }})\,=\,-\sqrt{\frac{4A}{\pi }}\frac{dA}{dt}$$

where *V* is the volume of the GUV, where it is assumed that the GUV is spherical. Based on these geometrical conditions, the efflux for some example GUVs is calculated from the fitted area loss (Table 1 and Supplementary Figure S2). The efflux for the pure fluid-phase GUVs (*Q* > 1000 *μ*m^3^/s) is considerably higher than for the pure gel-phase GUVs (*Q* < 200 *μ*m^3^/s). Our estimations of the fluxes and the correlation to the formed pore sizes are very rough, based on the assumptions of equation (7) and the geometrical conditions in equation (8). Nevertheless, this large difference in the fluxes for the fluid-phase and gel-phase GUVs indicates that the formed pores in the fluid-phase GUVs are considerably larger than the formed pores in the gel-phase GUVs. This agrees with the observations of macropores for the binary systems. All binary GUVs have shown similar flux to the pure fluid-phase GUVs, independent of the DPPC percentage and its mixing behaviour. This indicates that the pores in binary GUVs have comparable pore sizes as in pure fluid-phase GUVs. Contrary to the expectations, the homogeneous-GUVs, where no phase separation is observed and which appear to have predominantly gel-phase properties, show similar pore sizes as the pure fluid-phase GUVs. This might be due to small fluid-phase domains in these GUVs, that cannot be resolved with fluorescence microscopy. Consequently, based on the experimental observations of macropores and the approximation of the effluxes, it can be concluded that the permeabilisation of the heterogeneous membranes is mainly determined by the presence of fluid-phase domains.

**Table 1:** The estimated values of *Q* for the different GUVs.

Pure DPhPC GUVs	2:8 DPPC:DPhPC GUVs	8:2 DPPC:DPhPC GUVs	Pure DPPC GUVs
*Q* > 1000 *μ*m^3^/s	*Q* > 1000 *μ*m^3^/s	*Q* > 1000 *μ*m^3^/s	*Q* < 200 *μ*m^3^/s

For the 8:2 DPPC:DPhPC GUVs, the homogeneous-GUVs have displayed a higher electrical stability than the domain-GUVs. This increased stability may be due to the predominant gel-phase of the lipids in the homogeneous-GUVs^27^. The domain-GUVs, on the other hand, also contain fluid-phase domains. Consequently, the lipid loss takes place at a lower electric field, due to the presence of fluid-phase domains. However, it must be noted that the different mixing behaviour could also be a consequence of a different membrane tension of the GUVs^30^. These two parameters, gel-phase lipid ratio and membrane tension, are poorly controlled due to the used method for the preparation of the GUVs and can both cause an increased stability of the GUVs.

Finally, we need to comment on the slow post-pulse shrinkage of the fluid-phase GUVs. This slow post-pulse shrinkage is always observed in combination with a complete contrast loss after one of the individual pulses. Therefore, this shrinkage effect is linked to long-lived defects caused by the electric pulse. Polak *et al*.^14^ have already shown that the properties of the carbon-chain determine the electrical stability of the membrane. In this research we have used DPhPC lipids, which contain methyl-branched carbon-chains. These lipids are known to have slower lateral diffusion than the often used unsaturated DOPC^43^. It is plausible that this nature of the DPhPC lipids slows down the resealing of the created pores. The slow contrast loss indicates the presence of an influx of glucose molecules, whereas the shrinkage indicates an efflux of the inner liquid. Therefore, it can be concluded that during the resealing of the membrane both an influx and efflux are present, where the net flux is directed outward. It seems plausible that the post-pulse shrinkage of the GUVs can be caused by the shear stress on the membrane due to this efflux, which is driven through the pores by the post-pulse membrane tension.

## Conclusion

To elucidate the role of gel-phase lipids in electroporation of the cell membrane, we have studied GUVs composed of DPPC and DPhPC lipids, as well as GUVs composed of binary mixtures of both lipids. Our observations show that pure fluid-phase GUVs shrink after application of an electroporative electric pulse due to the formation of vesicles and/or tubules. On the contrary, pure gel-phase GUVs buckle permanently upon electroporation without any detectable lipid loss. The electric field can act to expel the lipids from fluid-phase GUVs in the form of tubules and vesicles, but not from gel-phase GUVs, due to the much higher surface viscosity of the gel-phase lipids. Likewise, it is observed that the fluid-phase lipids are removed by the electric field from the binary GUVs, while the remaining gel-phase lipids induce buckling of the GUVs. The critical electric field required to observe lipid loss increases with increasing percentage of DPPC lipids. Moreover, both binary systems have revealed macropore formation. Finally, we have analysed the post-pulse efflux from the GUVs, to estimate the sizes of the experimentally observed pores. The estimates suggest that the pores are comparable to the pores of the pure fluid-phase GUVs, showing the ability to form large pores despite a high percentage of gel-phase lipids.

These results provide an insight on the role of the different domains in the membrane of living cells during electroporation. On the one hand, the gel-phase lipids can provide an increased electrical stability to the membrane, depending on the mixing behaviour of the two phases. On the other hand, it seems that the transport across the cell membrane is mainly determined by the fluid-phase domains and is not considerably affected by the presence of gel-phase domains.

## Methods

### GUV preparation

The GUVs were prepared from 1,2-diphytanoyl-sn-glycero-3-phosphocholine (DPhPC), 1,2-dipalmitoyl-sn-glycero-3-phosphocholine (DPPC) and 1,2-dioleoyl-sn-glycero-3-phosphoethanolamine-N-(lissamine rhodamine B sulfonyl) (ammonium salt) (DOPE-RhoB), all purchased from Avanti Polar Lipids, Inc. All lipids were dissolved in chloroform (Sigma Aldrich), 1 mg/ml, prior to use. Afterwards, the lipids were mixed in the correct ratio: 99% vol DPPC or DPhPC and 1% vol DOPE-RhoB for the pure phases and for the mixtures 79.5% vol DPPC or DPhPC, 19.5% vol DPhPC or DPPC and 1% vol DOPE-RhoB.

The GUVs were prepared by use of the electroswelling technique. The lipid mixtures were deposited on two indium tin oxide (ITO) slides (Sigma Aldrich), 20 *μ*L lipid solution on each slide. The slides were placed in a teflon holder facing each other with a 1.5 mm distance, submerged in 1 mL of 200 mM sucrose (Amresco). It was assured that the swelling took place above the transition temperature of the lipids (for pure DPhPC at room temperature, for all lipid mixtures that contained DPPC at 57 °C). An alternating field was applied of 1 V and 10 Hz for 1 hour, and subsequently changed to 1.5 V and 5 Hz for 2 hours, based on the procedure of Knorr *et al*.^19^. After the swelling, the GUV solution was gradually cooled down (~0.5 °C/min). It was assumed that the low cooling rate assures that the lipids organise in the energetically most favoured formation^30^. Afterwards, the GUV solution was diluted at least 10 times with 200 mM glucose (Sigma Aldrich). By using the sucrose interior and a glucose exterior of the GUVs, a contrast difference was observed. Only GUVs with a proper contrast difference and a spherical shape were selected for these experiments, to assure that the membranes were defect free. Additionally, the spherical shape of GUVs indicated that all GUVs in our experiments had a nonzero initial tension. Finally, we have observed GUVs that have shown membrane fluctuations long after the pulse and occasionally even disintegrated. Since these responses were long after the pulse, we assumed that these phenomena may be induced by different causes, these GUVs were excluded from the analysis.

### Microscopy

All microscopy bright field experiments were conducted with the inverted fluorescence microscope (Zeiss Axio Observer.Z1) and captured by the Andor iXon3 camera, using a 40× oil immersion objective (Ph3, Plan-Neofluar, 40×/1.30 Oil). The contrast of the images was enhanced by optimising the aperature diaphragm before imaging and adjusting the histogram during processing of the images. The fluorescent images were captured by using the Texas Red filter (45 TR, EX BP 560/40, BS FT 585, EM BP 630/75). The confocal experiments were conducted on the Zeiss LSM 710 inverted confocal microscope, using a 40× oil immersion objective (Fluar, 40×/1.30 oil, M27) and the HeNe-laser (543 nm) to image the GUVs.

### Electroporation setup

For the electroporation experiments, custom-made stainless steel electrodes were used with two fixed distances of 0.6 and 1 mm (Fig. 1A–C). Prior to the experiments, the electrodes were submerged in 1 mL of 200 mM glucose solution. 20 *μ*L of freshly prepared GUVs was deposited in between the electrodes. Subsequently, electric pulses with duration of 500 *μ*s were applied. The pulse length was comparable to the characteristic time in which the transmembrane voltage builds on the GUV membrane in our experimental conditions. This charging time *τ* is estimated using equation (4) with a membrane capacitance of 0.5–0.6 *μ*F/cm^2^ ^44^ and 0.45 *μ*F/cm^2^ ^19^ for the fluid-phase and gel-phase lipids, respectively, and 4.5 *μ*S/cm^33^ and 6 *μ* S/cm^33^ for the external and the internal conductivity, respectively; for a GUV with typical radius of 20 *μ*m *τ* is about 300 *μ*s (fluid-phase GUV) or 250 *μ*s (gel-phase GUV). The time interval between application of individual pulses was approximately 5 minutes, to minimize the effect of the former pulse. With each subsequent pulse, the pulse amplitude was gradually increased in steps by 10 to 150 V/mm. Before, during, and after application of each pulse, a time lapse of the response of the GUVs was captured by imaging at approximately 10 to 25 frames per second. For each applied pulse, a separate movie was recorded. After the pulse application, the GUVs were imaged until relaxed back to stable shape, which meant that the fluid-phase lipids were not floppy at the end of the time lapse. To capture the mechanism of lipid loss, electroporation epxeriments at the confocal microscope were conducted. The pulse specifications were identical to the bright field experiments, and the images were taken at 1.6 frames per second. In addition, we applied multiple 5 ms pulses of 0.33 Hz to compare the lipid loss mechanism to former research^16^. The joule heating (less than 0.06 K) and evaporation in order to increase the exterior osmotic pressure (in the order of 20 mOsm) during the experiments are negligible, details can be found in supplementary Sections S5 and S6. All the data is available from the corresponding author.

### Data analysis

The area *A* and perimeter *P* of the GUVs were tracked by a custom-made Matlab script, as shown in Fig. 1D, by use of an edge detection. The perimeter and the area of the GUV were determined by a pixel count for most frames of the movie. Subsequently, the area was normalised over the area prior to the given pulse (*A*_*norm*_ = *A*_*f*_/*A*_*i*_). Additionally, the form factor of the GUVs during the movie was calculated $(FF=4\pi A/{P}^{2})$. Subsequently, the final area and form factor (at time ~5 min after pulse application) were plotted as a function of electric field per GUV. Afterwards, the mean of both factors of all GUVs was calculated with a bin size of the electric fields of 30 V/mm. Due to the non-spherical and asymmetrical shape of the GUVs after exposure to multiple pulses, the normalised area and the form factor were plotted against the applied electric field and not the induced transmembrane voltage.

## Electronic supplementary material

Supplementary Movie S1

Supplementary Movie S2

Supplementary Movie S3

Supplementary Movie S4

Supplementary Movie S5

Supplementary Movie S6

Supplementary information
