# Supplementary material for: The role of gel-phase domains in electroporation of vesicles
Source: Sci Rep. 2018 Mar 19;8:4758. doi: 10.1038/s41598-018-23097-9 (PMC5859178; doi:10.1038/s41598-018-23097-9)
Supplement: Supplementary file 7 — Supplementary information [file 41598_2018_23097_MOESM7_ESM.pdf]

Supplementary information for

The role of gel-phase domains in electroporation of vesicles

Dayinta L. Perrier[1], Lea Rems[1], Michiel T. Kreutzer[1], and Pouyan E. Boukany[1,*]

[1]Department of Chemical Engineering, Delft University of Technology, 2629 HZ Delft, The Netherlands
[*]Correspondence and requests for materials should be addressed to P.E.B (email: P.E.Boukany@tudelft.nl)

Table of Contents

S1 Movie captions 2
S2 Lipid loss in DPhPC fluid-phase GUVs and buckling DPPC gel-phase GUVs 3
S3 Calculation of the efflux for the GUVs exposed to the electric pulses 4
S4 Joule heating during electroporation of vesicles 4
S5 Evaporation of the exterior liquid during experiments 5
References 6

S1 Movie captions

Supplementary Movie S1. The response of a DPhPC fluid-phase GUV to the third pulse of 500 µs at 97 V/mm. The GUV is imaged in the bright field. The movie is speed up 5.4 times. Observation period: 208 seconds. The bright spots on the GUV surface are possibly small vesicles that have been present from the start (before applying pulses). No increase in the GUV surface area is observed, for which it is assumed that these small vesicles do not influence the surface area of the GUV.

Supplementary Movie S2. Electroporation of a DPPC gel-phase GUV during the third 500 µs pulse at 595 V/mm. The GUV is imaged in the bright field. The movie is speed up 2.5 times. Observation period: 153 seconds.

Supplementary Movie S3. Buckling of a DPPC gel-phase GUV during the fourth 500 µs pulse at 744 V/mm. The GUV is imaged in the bright field. The movie is speed up 3.8 times. Observation period: 262 seconds.

Supplementary Movie S4. The response of a binary GUV with 20 mol% DPPC and 80 mol% DPhPC lipids to the third 500 µs pulse at 29 V/mm. Before and after the pulse, the GUV is imaged in the fluorescence mode to capture the response of the domains. During pulse application, the GUV is imaged in the bright field. The movie is speed up 6.3 times.

Supplementary Movie S5. The response of the homogeneous-GUV with 80 mol% DPPC and 20 mol% DPhPC lipids to the fourth 500 µs pulse at 890 V/mm. Before and after the pulse, the GUV is imaged in the fluorescence mode to capture the response of the domains. During pulse application, the GUV is imaged in the bright field. The movie is speed up 3.4 times.

Supplementary Movie S6. The response of the domain-GUV with 80 mol% DPPC and 20 mol% DPhPC lipids to the first 500 µs pulse at 445 V/mm. Before and after the pulse, the GUV is imaged in the fluorescence mode to capture the response of the domains. During pulse application, the GUV is imaged in the bright field. The movie is speed up 2.2 times.

S2 Lipid loss in DPhPC fluid-phase GUVs and buckling DPPC gel-phase GUVs

To demonstrate lipid loss in fluid-phase GUVs and lack thereof in gel-phase GUVs, confocal images of the pure fluid-phase and pure gel-phase GUVs are captured after an individual 500 µs pulse (Figure S1A and B). Tubular and vesicular protrusions are observed in a DPhPC fluid-phase GUV, whereas neither tubulation nor vesicle formation is observed for a DPPC gel-phase GUV.

We have exposed the GUVs to multiple 5 ms pulses following Portet *et al*.[1] As observed before, the lipid loss and associated shrinkage in the fluid-phase GUVs is shown to be more pronounced when multiple 5 ms pulses are applied, compared to the individual 500 µs pulses (Figure S1C). The DPPC gel-phase GUVs do not demonstrate lipid loss, despite the longer pulse duration, whereas a buckling effect has been observed for these pulse parameters (Figure S1D). These results give an additional argument that the viscosity of the gel-phase lipids hinders lipid expel in gel-phase GUVs.

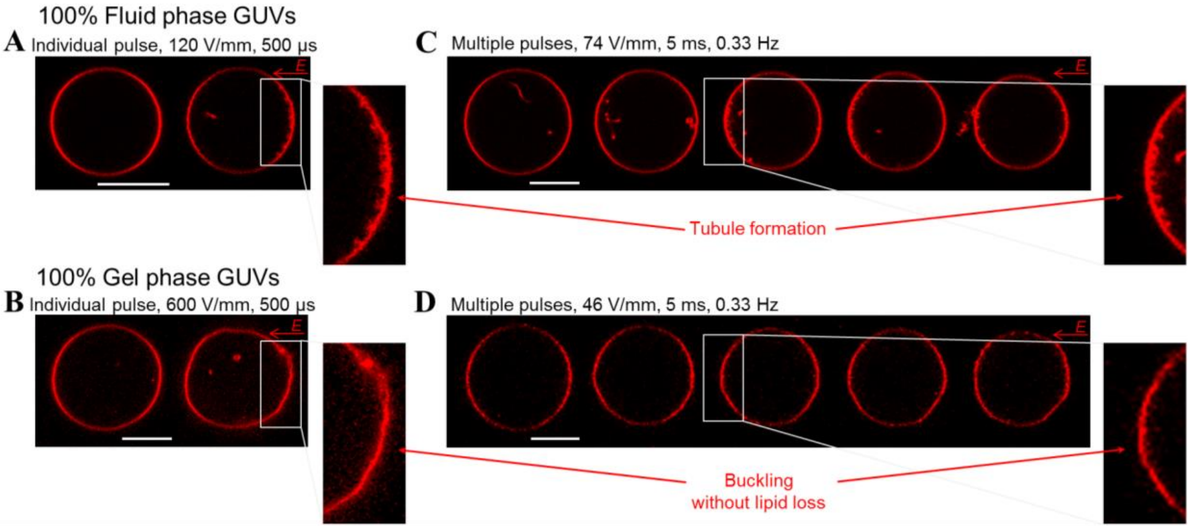

Figure S1: Confocal images of the pure fluid-phase and gel-phase GUVs exposed to electric pulses. (A) A pure fluid-phase GUV exposed to an individual 500 µs pulse of 120 V/mm. The lipids in the middle of the GUV have already been detected prior to pulse application, and thus are not indicating lipid expel. The tubules on the right side of the GUV are caused by the electric pulse, indicating lipid expel. (B) A pure gel-phase GUV exposed to an individual 500 µs pulse of 600 V/mm. The lipids in the middle of the GUV have already been detected prior to pulse application, and thus are not indicating lipid expel. (C) A pure fluid-phase GUV exposed to multiple 5 ms pulses, to enhance the lipid loss effect. (D) A pure gel-phase GUV exposed to multiple 5 ms pulses, to enhance the lipid loss effect. This shows that indeed no tubules nor vesicles are formed due to the pulse, even during the application of longer electric pulses. The scale bar in all images is 10 µm.

S3 Calculation of the efflux for the GUVs exposed to the electric pulses

The effluxes for the GUVs are calculated from the change in area during the electroporation experiments. The area of the GUVs is captured and tracked over time. This data is fitted, in order to calculate the flux of the GUVs as a function of time, as shown in Figure S5 (A-D).

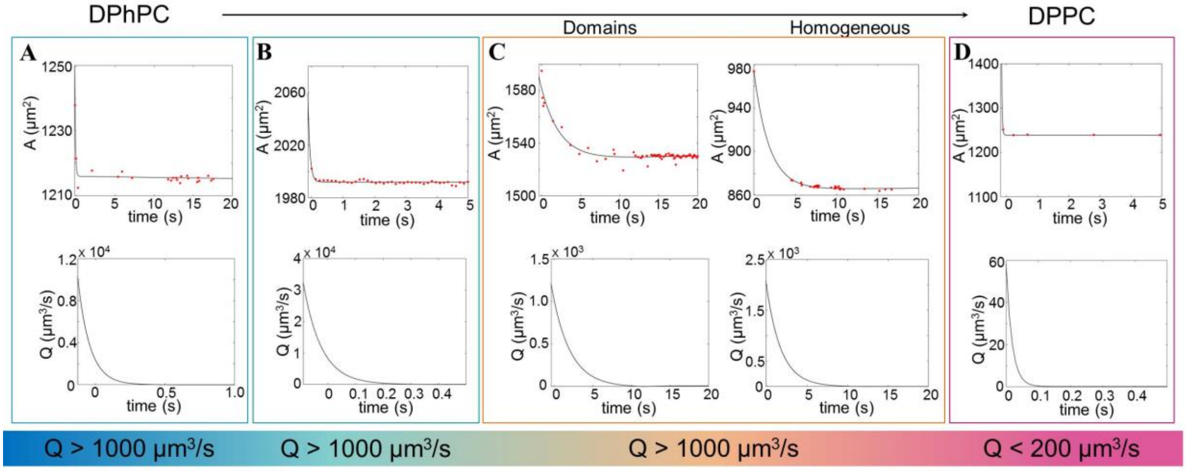

Figure S2. The absolute area of the different GUVs as a function of time: (A) pure fluid-phase lipids (R-square: 0.95), (B) 2:8 DPPC:DPhPC (R-square: 0.96), (C) 8:2 DPPC:DPhPC (domain-GUVs R-square: 0.93 and homogeneous-GUVs R-square: 0.98), and (D) pure gel-phase lipids (R-square: 0.98). The data from the experiments are depicted in red dots and the solid black lines represent the fitted area and subsequently the calculated flux from the fitted area.

S4 Joule heating during electroporation of vesicles

To ensure that the gel-phase GUVs remained in gel phase during the experiments and did not undergo phase transition due to the Joule heating from the pulse application, we calculate the maximum temperature increase during application of a 500 µs pulse. Considering that the delivered heat Q equals the work done by the electric field W

$$Q = c_m m \Delta T$$

$$W = JE\Delta t = \lambda_e E^2 t_{pulse}$$

$$\Delta T = \frac{W}{c_m m} = \frac{E^2 \lambda_e}{c_m \rho} t_{pulse}$$

where ΔT is the temperature rise (in kelvins), c_m is the heat capacity of the sample, m is the mass of the sample, J is the electric current density, E is the electric field intensity, t_{pulse} is the pulse duration, λ_e is the electrical conductivity of sample, and ρ is the density of the sample.

For an electric field of 1000 V/mm (the maximum electric field used in experiments):

$$\Delta T = \frac{E^2 \lambda_e}{c_m \rho} t_{pulse} = \frac{(10^6\ \mathrm{V/m})^2 \times 5 \cdot 10^{-4}\ \mathrm{S/m}}{4200 \frac{\mathrm{J}}{\mathrm{kgK}} \times 1000\ \mathrm{kg/m^3}} \times 5 \cdot 10^{-4}\ \mathrm{s} = 0.06\ \mathrm{K}$$

The heat capacity and density are taken for water. The electrical conductivity of 200 mM glucose solution is taken from Riske and Dimova[2]. The calculated temperature increase is too low to have any effect on the lipids.

S5 Evaporation of the exterior liquid during experiments

Some of the fluid-phase DPhPC GUVs have exhibited a slow (on the timescale of several seconds) and profound (~ 20%) size decrease after the pulse application, which has not been reported before for GUVs prepared from other fluid-phase lipids, such as egg phosphatidylcholine or DOPC[1-4]. We have checked whether this phenomenon can be due to an increase in the exterior osmotic pressure caused by the evaporation of the exterior liquid.

The evaporation of the exterior liquid during the experiments has been determined by a control experiment. The imaging chamber has been filled with 1.1 ml of 200 mM glucose solution and placed under the microscope, similarly as during the experiments. The weight of the glucose solution has been determined every hour for three hours, to find the total weight loss of the glucose solution. It must be noted that most experiments have taken less than two hours; however, in order to ensure that no side effects of evaporation have been imaged, we have monitored the evaporation for three hours. In one hour, approximately 3.5% volume has been evaporated, and thus approximately 10% of the total volume has been evaporated in three hours. The 10% evaporated volume can increase the exterior osmotic pressure by ~ 10 mOsm. We do not expect such small increase in the osmotic pressure to considerably affect our results, since an osmotic difference of ~ 20 mOsm (being higher in the exterior with respect to the interior) is often used before in electroporation experiments on fluid-phase GUVs[1,3]. Furthermore, GUVs can exhibit a similar slow size decrease, if they have been immediately exposed to a single high-intensity electric pulse, before any considerate evaporation can take place (not shown). Therefore, we attribute this slow size decrease, not reported before for other fluid-phase lipids, to the nature of the DPhPC lipids.

References

1 Portet, T. *et al.* Visualization of Membrane Loss during the Shrinkage of Giant Vesicles under Electropulsation. *Biophysical Journal* **96**, 4109-4121 (2009).

2 Riske, K. A. & Dimova, R. Electro-Deformation and Poration of Giant Vesicles Viewed with High Temporal Resolution. *Biophysical Journal* **88**, 1143-1155, doi:10.1529/biophysj.104.050310 (2005).

3 Mauroy, C. *et al.* Giant lipid vesicles under electric field pulses assessed by non invasive imaging. *Bioelectrochemistry* **87**, 253-259, doi:10.1016/j.bioelechem.2012.03.008 (2012).

4 Portet, T. & Dimova, R. A New Method for Measuring Edge Tensions and Stability of Lipid Bilayers: Effect of Membrane Composition. *Biophysical Journal* **99**, 3264-3273 (2010).
